# Effects of Charge Transport Materials on Blue Fluorescent Organic Light-Emitting Diodes with a Host-Dopant System

**DOI:** 10.3390/mi10050344

**Published:** 2019-05-25

**Authors:** Neng Liu, Sijiong Mei, Dongwei Sun, Wuxing Shi, Jiahuan Feng, Yuanming Zhou, Fei Mei, Jinxia Xu, Yan Jiang, Xianan Cao

**Affiliations:** 1 Hubei Key Laboratory for High-efficiency Utilization of Solar Energy and Operation Control of Energy Storage System, Hubei University of Technology, Wuhan 430068, China; 13618655764@163.com (N.L.); meisijiong@163.com (S.M.); sdwwant@163.com (D.S.); 18971141948@163.com (W.S.); 17764283212@163.com (J.F.); xujx@mail.hbut.edu.cn (J.X.); yanjiang5909@126.com (Y.J.); 2 Department of Computer Science and Electrical Engineering, West Virginia University, Morgantown, WV 26506, USA; xacao@mail.hbut.edu.cn

**Keywords:** blue organic light emitting diodes, transport materials, host-dopant

## Abstract

High efficiency blue fluorescent organic light-emitting diodes (OLEDs), based on 1,3-bis(carbazol-9-yl)benzene (mCP) doped with 4,4’-bis(9-ethyl-3-carbazovinylene)-1,1’-biphenyl (BCzVBi), were fabricated using four different hole transport layers (HTLs) and two different electron transport layers (ETLs). Fixing the electron transport material TPBi, four hole transport materials, including 1,1-Bis[(di-4-tolylamino)phenyl]cyclohexane (TAPC), N,N’-Di(1-naphthyl)-N,N’-diphenyl-(1,1’-biphenyl)-4’-diamine(NPB), 4,4’-Bis(N-carbazolyl)-1,1,-biphenyl (CBP) and molybdenum trioxide (MoO_3_), were selected to be HTLs, and the blue OLED with TAPC HTL exhibited a maximum luminance of 2955 cd/m^2^ and current efficiency (CE) of 5.75 cd/A at 50 mA/cm^2^, which are 68% and 62% higher, respectively, than those of the minimum values found in the device with MoO_3_ HTL. Fixing the hole transport material TAPC, the replacement of TPBi ETL with Bphen ETL can further improve the performance of the device, in which the maximum luminance can reach 3640 cd/m^2^ at 50 mA/cm^2^, which is 23% higher than that of the TPBi device. Furthermore, the lifetime of the device is also optimized by the change of ETL. These results indicate that the carrier mobility of transport materials and energy level alignment of different functional layers play important roles in the performance of the blue OLEDs. The findings suggest that selecting well-matched electron and hole transport materials is essential and beneficial for the device engineering of high-efficiency blue OLEDs.

## 1. Introduction

Organic light-emitting diodes (OLEDs) are constructed with several organic or inorganic layers between the anode and cathode, and have attracted great interest throughout the world owing to their advantages, such as fast response time, high contrast ratio, wide view angle, and low power consumption [1,2,3,4,5,6,7,8,9,10]. In addition, their remarkable ability to fabricate on flexible substrates is the most significant characteristic that facilitates the fabrication of OLED displays with various shapes and sizes, which is hardly implemented by using other existing technologies [5]. High-performance OLEDs with primary RGB colors are essential for developing high-quality full-color displays and white-light emission. However, state-of-the-art blue OLEDs have relatively poor emission performance compared with other red and green counterparts, in terms of luminous efficiency, color purity, and operational lifetime. Therefore, a lot of efforts have been made to improve the emission performance of blue OLEDs. It is widely accepted that a guest–host system is a useful method to improve emission characteristics of blue devices [4,6,11,12]. However, most of the research has been focused on the type and concentration of dopants, with only a few studies concentrated on the cooperation of hole and electron transport layers. Several papers about blue fluorescent OLEDs based on a host doped with BCzVBi have been reported, and a suitable host is expected to enable efficient energy transfer to the guest, giving rise to efficient blue luminescence [11,12]. 

Many efforts have been made to discuss the effects of hole and electron transport materials on the characteristics of OLED devices [13,14,15,16,17,18,19,20]. Jou et al. reported that exciton recombination and electric field distribution across the emission layer can be affected by charge transport materials, which play important roles in the luminance and turn-on voltage of devices [14]. Liu et al. [15] discussed the impact of electron transport materials on devices’ stability, and Giebeler et al. [16] compared the effects of different hole transport materials on the emission characteristics. Although several groups have studied the effect of electron and hole transport materials on the performance of blue OLED devices, including efficiency and lifetime, quite a few of them systematically investigated the selection of appropriate hole transport material (HTM) and electron transport material (ETM) and analyzed the limiting factors to fabricate high-efficiency OLED devices with low turn-on voltage.

The energy levels and charge carrier mobility of charge transport materials are intimately related to the efficiency and operating voltage of devices. Since efficient injection of charges into emission layer (EML) leads to a high space charge limited current flow that reduces the operating voltage, it is suggested that HTM and ETM should have a high charge carrier mobility [14]. Meanwhile, only one kind of carrier with high mobility is usually unfavorable for efficiency. The mobility of holes is often higher than that of electrons, which will reduce the recombination of holes and electrons, and thus OLED devices introducing ETM and HTM with nearly equivalent carrier mobility should be employed. Therefore, it is necessary to make a proper medium to obtain the desired current efficiency and turn-on voltage, as well as other properties, via the selection of appropriate HTM and ETM, which can facilitate the injection and balanced transport of holes and electrons.

In this paper, the influence of charge transport materials on the performance of bis(9- carbazolyl)benzene (mCP)-BCzVBi-based OLEDs was studied in detail, especially in the aspect of efficiency and working voltage. The luminous characteristics of these devices were measured and compared with each other. In addition, the behavior of holes and electrons was discussed in detail, to re-examine the relationship between charge transport materials and devices’ performance. From the above experiments and analysis, we hope that a more excellent performance of blue OLED devices can be obtained by selecting well-matched ETM and HTM materials.

## 2. Experiments 

**Figure 1:**
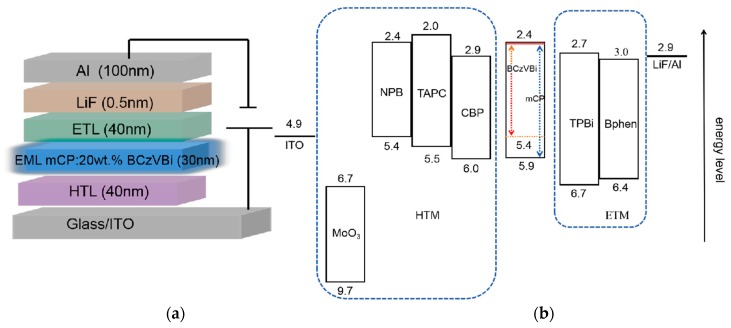
(**a**) The schematic structure of our blue organic light-emitting diodes (OLEDs). (**b**) The energy level diagram of the blue OLEDs.

Blue OLEDs with a mCP:20wt.% BCzVBi host–guest system were fabricated on pre-patterned commercial indium-tin-oxide (ITO) coated glass substrates, whose sheet resistance was around 15 Ω/□. Typically, the substrates were ultrasonically cleaned in acetone, methanol, and deionized water for 5 min sequentially. Then, the substrates were dried with a nitrogen gun and exposed to oxygen plasma for 5 min, which can effectively modify the work function of ITO. After that, the substrates were transferred to a physical vapor thermal evaporation system with a base pressure of ~3 × 10^−7^ Torr. All functional layers, including organic, inorganic, and metal materials, were deposited on unheated substrates with different rates less than 0.1 nm/s, which guaranteed the film quality. The blue OLEDs had a 30 nm thick light-emitting layer, comprising an mCP host doped with 20 wt.% BCzVBi, sandwiched between a 40 nm thick hole transport layer (HTL) and a 40 nm thick electron transport layer (ETL). In this paper, four kinds of HTLs (1,1-Bis[(di-4-tolylamino)phenyl]cyclohexane (TAPC), N,N’-Di(1-naphthyl)-N,N’-diphenyl-(1,1’-biphenyl)-4’-diamine (NPB), 4,4;-Bis(N-carbazolyl)-1,1,-biphenyl (CBP) and molybdenum trioxide (MoO_3_)) and two kinds of ETLs (2,2’,2″-(1,3,5-Benzinetriyl)-tris(1-phenyl-1-H-benzimidazole) (TPBi) and 4,7-Diphenyl-1,10-phenanthroline (Bphen)) were applied in our devices. Finally, a 0.5 nm thick lithium fluoride (LiF) and a 100 nm Al were used for the electron injection layer and cathode, respectively. Each substrate contained four devices, with an effective area of 0.1 cm^2^. Figure 1a shows the schematic structure of the blue OLED devices studied in this work. All fabricated samples were encapsulated with a glass lid in a N_2_-filled chamber and characterized at room temperature. Figure 1b illustrates the energy level diagram of blue OLEDs, showing the energy levels of the highest occupied molecular orbital (HOMO) and lowest unoccupied molecular orbital (LUMO) of organic materials, and the working functions of the two electrodes, which are referenced in the literature [4,5,6,7,8,9,10,11].

The current density–voltage (J–V) characteristics of OLEDs were tested using a Keithley 2400 source meter system and the electroluminescence (EL) spectra of the devices were measured by an Ocean Optics fiber-optic spectrometer. The photoluminescence (PL) spectra were measured by a HITACHI F-4600 luminescence spectrometer with an excitation wavelength of 320 nm. The luminance of the devices was measured by a Keithley 2000 multimeter, coupled with a calibrated silicon photodetector (1 cm in diameter), which was put directly onto the surface of an individual device, ensuring all photons emitted from the glass side were captured. Finally, in order to test the stability of the devices, as-fabricated OLEDs were stressed at a constant current density of 20 mA/cm^2^, and the voltage and luminance were collected with a time interval of 5 s. The OLED lifetime was determined according to the luminance evolution profile recorded at 20 mA/cm^2^.

## 3. Results and Discussion

**Figure 2:**
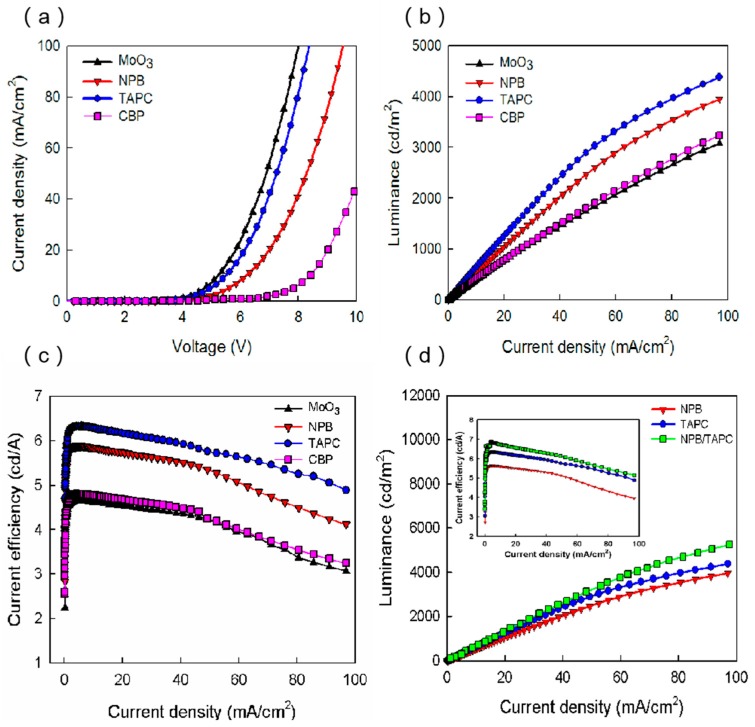
(**a**) Current density versus voltage curves, (**b**) luminance versus current density curves, (**c**) current efficiency versus current density curves of blue OLEDs with 2,2’,2″-(1,3,5-Benzinetriyl)-tris(1-phenyl-1-H-benzimidazole) (TPBi) electron transport layers (ETL) and four different hole transport layers (HTLs). (**d**) Luminance versus current density curves of blue OLEDs with TPBi ETL and N,N’-Di(1-naphthyl)-N,N’-diphenyl-(1,1’-biphenyl)-4’-diamine (NPB), 1,1-Bis[(di-4-tolylamino)phenyl]cyclohexane (TAPC), NPB/TAPC HTLs.

Firstly, the blue OLEDs with the structure of ITO/HTL/mCP:20 wt.% BCzVBi/TPBi/LiF/Al were fabricated and characterized, in which TAPC, NPB, CBP, and MoO_3_ were used for HTL. In the following, these four devices are labeled as TAPC device, NPB device, CBP device, and MoO_3_ device, for clarity. Figure 2a displays the current density–voltage (J–V) characteristics of blue OLEDs based on different HTMs. As shown in Figure 2a, of these four kinds of devices, the MoO_3_-based OLED had the lowest turn-on voltage of ~3.61 V, whereas the CBP-based OLED had the highest value of ~4.82 V. When the current density was set to be 40 mA/cm^2^, the operating voltages of the blue OLEDs based on TAPC, NPB, CBP, and MoO_3_ were 6.92 V, 7.96 V, 9.8 V, and 6.67 V, respectively. These characteristics can be explained by the hole injection from the ITO anode and the hole transport across the HTL. The former is usually related to the energy level alignment at the HTL/anode and HTL/EML interfaces, and the latter is mainly influenced by the hole mobility of HTMs. It can be seen in Figure 1b that holes injected from ITO to CBP must overcome a big energy barrier of ~1.1 eV at the ITO/HTL interface, while for the devices with NPB and TAPC HTLs, the energy barrier decreases to 0.5 eV and 0.6 eV, respectively. Thus, one can conclude that the NPB and TAPC devices have more efficient hole injection from ITO to HTL than the CBP device. As reported in other articles, the hole mobility of NPB, TAPC, and CBP are 8.8 × 10^−4^, 1 × 10^−2^, and 2 × 10^−3^ cm^2^·V^−1^·s^−1^, respectively [4,11,12,20]. Although the hole mobility of CBP is larger than that of NPB, the operating voltage of the CBP device is higher, suggesting that both hole mobility and energy level alignment play important roles in the J–V characteristics. For the CBP device, high hole mobility leads to high-efficiency hole transport, while the larger energy barrier impedes the hole injection, which finally causes the highest operating voltage among the NPB, TAPC, and CBP devices. In addition, the energy barrier of the TAPC device is 0.1 eV lager than that of the NPB device, while the hole mobility of TAPC is ~10 times higher than that of NPB, leading to the result that the operating voltage of the TAPC device is smaller than that of the NPB device. MoO_3_ is widely used as hole injection material. It is also an excellent hole transport material due to its high electron conductivity, good stability, and deep-lying energy levels [19]. As seen in Figure 1b, the energy levels of MoO_3_ lie below the HOMO levels of mCP and BCzVBi, which suggests that MoO_3_ can accept electrons from mCP and BCzVBi, generating free holes in the EML. This process is equivalent to hole injection and transport from the MoO_3_ layer to EML. Moreover, this hole injection and transport process are quicker and more efficient than other conventional devices using NPB, TAPC, and CBP HTLs. Thus, it is reasonable that the MoO_3_ device shows the best performance seen in the J–V characteristics.

Figure 2b shows the luminance of blue OLEDs as functions of current density. As seen in Figure 2b, the luminance of the TAPC, NPB, CBP, and MoO_3_ based OLEDs were 2955, 2558, 1875, and 1760 cd/m^2^ at 50 mA/cm^2^, respectively. The luminance is strongly related to the number of excitons generated in the EML, and thus the balance between electrons and holes in the EML is of vital importance [9]. The energy barrier between LiF/Al and TPBi is 0.2 eV and the electron mobility of TPBi is ~6.5 × 10^−5^ cm^2^·V^−1^·s^−1^ [19]. It can be seen that both of the MoO_3_ and CBP devices had similar L-J characteristics, in which the luminance was lower than other two devices, since the charge balance was worse. As mentioned above, the hole injection and transport from MoO_3_ to EML was very efficient, while the electron transport was relatively slow from TPBi to EML because of the low electron mobility. This may lead to the fact that the number of holes reaching EML is larger than that of the electrons, causing seriously unbalanced holes and electrons, and correspondingly the lower luminance and efficiency. Similarly, the low luminance of the CBP device can be explained by the unbalanced holes and electrons in EML, which originated from the gap between the carrier mobility of CBP (2 × 10^−3^ cm^2^·V^−1^·s^−1^) and TPBi (~6.5 × 10^−5^ cm^2^·V^−1^·s^−1^). In addition, the low-efficiency hole injection of the CBP device caused by the large energy levels also led to the small number of holes in EML, which was related to the low luminance. For the NPB device, since NPB and TPBi have the similar carrier mobility of ~10^−5^–10^−4^ cm^2^·V^−1^·s^−1^, the NPB device can achieve well-balanced holes and electrons in the EML, which could generate more excitons and lead to a higher luminance [15,19]. However, the TAPC device with the large gap between the carrier mobility of TAPC (1 × 10^−2^ cm^2^·V^−1^·s^−1^) and TPBi (~6.5 × 10^−5^ cm^2^·V^−1^·s^−1^) showed a higher luminance than the NPB device, which does not correlate well to the above reasoning. It is noted that, for the TAPC device, the LUMO levels of mCP and BCzVBi were both 2.4 eV, which is 0.4 eV lower than TAPC. Therefore, there was effective electron blocking at the EML/TAPC interface which is beneficial to the recombination of excitons and the balanced carriers in EML [4]. However, the LUMO level of NPB was well aligned with mCP and BCzVBi, and electrons in EML can escape from EML to NPB, which ultimately reduced the effective number of excitons. 

Figure 2c illustrates the current efficiency–current density characteristics based on the four different HTMs. When the current density was 50 mA/cm^2^, the current efficiencies of the TAPC, NPB, CBP, and MoO_3_ devices were 5.75, 5.04, 3.78, and 3.56 cd/A, respectively. This result is consistent with the luminance discussed above, both indicating that the devices’ efficiencies are proportional to the number of excitons generated in the EML region, which depend on the balance between the electrons and holes in the EML. On increasing the current density, the luminance (or the number of excitons) increased rapidly, leading to an increase in the current efficiency. Afterward, the luminance increased more slowly with a further increase in the current density, which caused the decrease in the current efficiency, namely the efficiency roll-off. As reported, the efficiency roll-off of fluorescent doped OLEDs is mainly attributed to charge imbalance, while quenching processes appear to have only a minor role [13,21,22,23,24]. Therefore, the higher current efficiency in the TAPC device can be attributed to the balance of the number of electrons and holes in the EML, due to excellent hole injection and transport into the EML.

In LEDs, the current efficiency of devices is proportional to the value of external quantum efficiency (EQE) defined by the equation: EQE = *η*_out_*rqγ*, where *η*_out_ is the fraction of emitted photons that are coupled out of the device, *r* is the fraction of excitons that can potentially radiative decay, *q* is the photoluminescence quantum yield (PLQY) of emitters, and *γ* is the charge balance (*γ*≤1) [25,26]. Generally, the internal operation of LEDs does not influence *η*_out_, while *r* and *q* would be set with emitters. Hence, from the perspective of device engineering, the change of current efficiency with different HTLs is most sensitive to *γ*. In terms of charge mobility, both of the MoO_3_ and CBP devices exhibit the lowest efficiency since their charge balance is the worst among four devices, namely the hole mobility of MoO_3_ and CBP HTLs is much higher than the electron mobility of TPBi ETL. While the NPB device exhibits the medium efficiency since NPB and TPBi have the similar carrier mobility of ~10^−5^–10^−4^ cm^2^·V^−1^·s^−1^. The TAPC device exhibits the highest efficiency, which does not correlate well to the above reason. It is suggested that the other two factors affecting the balance of carriers, including the low contact barrier energy and effective electron blocking at HTL/EML interface, lead to the high efficiency of the TAPC device. As reported in another literature, the hole and electron mobility of the mCP host is 1.2 × 10^−4^ and 4 × 10^−5^ cm^2^·V^−1^·s^−1^, respectively. [27] The different charge mobilitis of the host may mainly affect the location and width of the recombination zone, governing the behaviors of charge injection and transport in the EML. [28,29] 

In order to examine that the effective electron blocking can extend the luminance, we inserted 10 nm TAPC to the NPB/EML interface in the original NPB device. The L-J characteristics of the NPB, TAPC, and NPB/TAPC devices are displayed in Figure 2d. As can be seen, the NPB/TAPC device showed the highest luminance of 3354 cd/m^2^ at 50 mA/cm^2^, which is higher than those of the NPB and TAPC devices, suggesting the favorable effect of electron blocking caused by TAPC. The inset in Figure 2d is the corresponding current efficiency as functions of current density. Higher current efficiency in the NPB/TAPC device can be attributed to the balance of the number of electrons and holes caused by the effective electron blocking [4]. 

**Figure 3:**
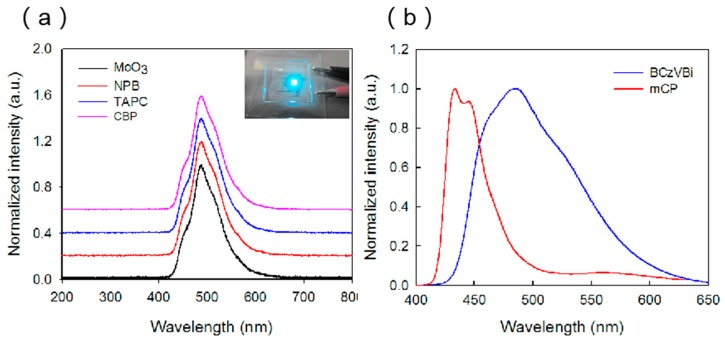
(**a**) Normalized electroluminescence spectra of OLEDs with TPBi ETL and four different HTLs. The inset is a luminescence image of the TAPC device at 50 mA/cm^2^. (**b**) Photoluminescence (PL) spectra of 30 nm mCP and BCzVBi films deposited on ITO substrates.

The normalized electroluminescence (EL) spectra of the blue OLEDs with different HTLs are displayed in Figure 3a. All the devices have a main blue peak at ~485 nm and a shoulder peak at ~445 nm, which indicate the existence of the energy transfer from host material to the guest dopant molecule. The photoluminescence (PL) spectra of the mCP and BCZVBi films were also measured, as shown in Figure 3b. The PL peak of BCzVBi is at ~485 nm and the PL peaks of mCP are at ~445 nm and 433 nm, which is consistent with the EL results. In the EL spectra, the main peak at ~485 nm can be attributed to the radiation transition of BCzVBi excitons which are transferred from mCP, while the shoulder peak at ~445 nm originates from the mCP excitons. In addition, the shapes of the EL spectra do not change as we change the HTM, indicating that the behavior of holes in HTL does not influence the blue emission.

**Figure 4:**
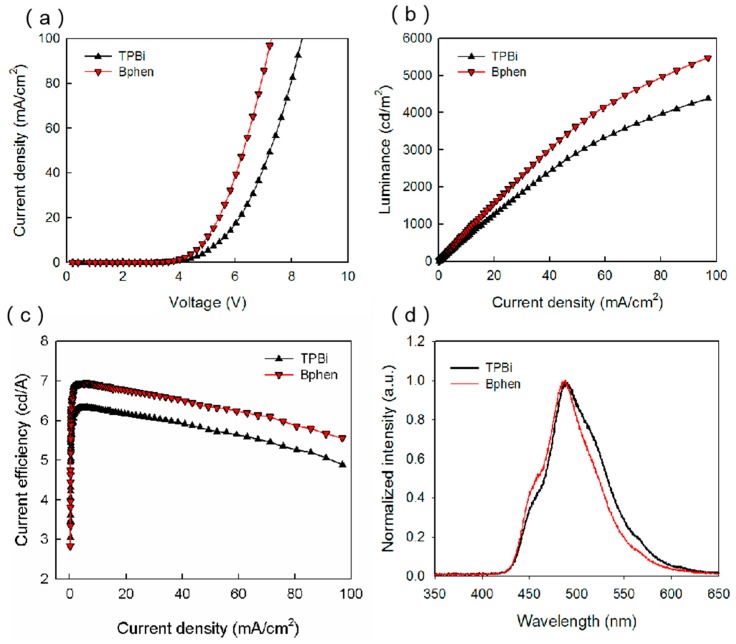
(**a**) Current density versus voltage curves, (**b**) luminance versus current density curves, (**c**) current efficiency versus current density curves, (**d**) normalized EL spectra of blue OLEDs with TAPC HTL and 4,7-Diphenyl-1,10-phenanthroline (Bphen), TPBi ETLs.

In order to further improve the balance of carriers in EML, another ETL with higher electron mobility was introduced. As reported in another paper, the electron mobility of Bphen is 3.3 × 10^−4^ cm^2^·V^−1^·s^−1^, which is ~10 times higher than that of TPBi [19]. The Bphen devices were also fabricated and characterized in order to study the role of ETL on the balanced transport. As discussed above, one can find that the TAPC-based OLED shows a better luminous performance than the other three kinds of devices. So, fixing the hole transport material TAPC, the blue OLEDs with Bphen and TPBi ETLs were compared and discussed. The device structure was ITO/TAPC/mCP:20wt.% BCzVBi/ETL/LiF/Al, which are labeled as the Bphen device and TPBi device for clarity. Figure 4a displays the current density–voltage characteristics of the devices with different ETLs. It can be seen that the Bphen device had a turn-on voltage of ~3.4 V, which was smaller than that of the TPBi device. The LUMO level of Bphen was 3 eV, which is 0.3 eV lower than that of TPBi, and thus the electron injection from cathode to Bphen was much easier. Moreover, the transmission of electrons in Bphen will be faster than that in TPBi because of its high electron mobility. Figure 4b illustrates the luminance–current density characteristics of the Bphen device and the TPBi device, whose luminance were 3640 and 2955 cd/m^2^, respectively. These results suggest that the carrier balance in EML was improved by substituting TPBi with Bphen. The hole mobility of TAPC (1 × 10^−2^ cm^2^·V^−1^·s^−1^) was much larger than the electron mobility of TPBi (6.5 × 10^−5^ cm^2^·V^−1^·s^−1^), resulting in more holes than electrons in EML. Bphen (3.3 × 10^−4^ cm^2^·V^−1^·s^−1^) had a higher electron mobility than that of TPBi, increasing the number of electrons in EML, and finally improving the balance of holes and electrons in EML. This helps to generate more excitons, which increases the efficiency of the devices. Figure 4c illustrates the current efficiency–current density characteristics of the two devices, which are consistent with the luminance curves. Figure 4d shows the normalized EL spectra of the two devices. The main peak at ~485 nm and the shoulder peak at ~445 nm can be attributed to the BCzVBi excitons and mCP excitons, respectively. It is noted that the strength of the shoulder peak increases slightly, which may be caused by the shift of recombination zoom to the HTL/EML interface because of the high electron mobility.

**Figure 5:**
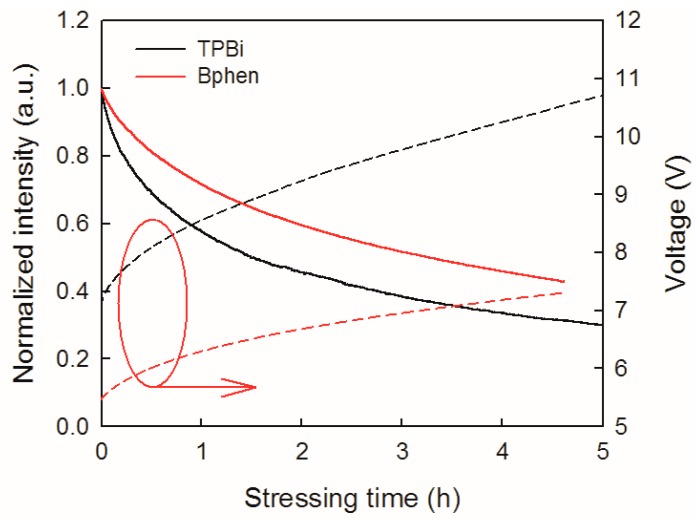
Evolution of the normalized luminance and voltage of blue OLEDs with Bphen and TPBi ETL stressed under 20 mA/cm^2^.

Figure 5 displays the evolution of the normalized luminance and operating voltage of the blue OLEDs with Bphen and TPBi ETLs at 20 mA/cm^2^. The luminance of these two devices exhibited similar stretched exponential decay. The average values of half-life of the Bphen and TPBi devices were tested to be 3.22 h and 1.55 h, respectively. Clearly, the blue OLED with the Bphen ETL is relatively reliable under stressing, and has a lifetime nearly two times longer than that with TPBi ETL. With the decrease in luminance, the operating voltage increases, and both the rates of luminance decay and voltage rise in the Bphen device are much slower. These results illustrate that ETM has an important impact on the OLED reliability, including the intrinsic stability of electron transport materials, the LUMO level, and electron mobility [15,19]. Although TPBi (Tg = 127 °C) [18] has a higher glass transition temperature than Bphen (Tg = 66 °C) [12], the Bphen device exhibited a more stable performance than the TPBi device. There is a fact that Bphen has higher electron mobility than that of TPBi and better energy level alignment with the working function of the Al cathode, which facilitate the high-efficiency injection and transport of electrons. For the TPBi device, the misalignment of the TPBi LUMO level with the working function of the Al cathode resulted in an energy barrier as well as an additional voltage drop at the ETL/cathode interface, which would lead to localized joule heating and accelerate electrochemical reactions [19]. Moreover, lower electron mobility means that a higher voltage drop across the ETL is needed for charge transport. These reasons may be responsible for the faster degradation of the TPBi OLED relative to the Bphen device.

The lifetime of our devices is higher than those of some phosphorescent OLEDs with the mCP host because of the better stability of fluorescent materials [18,29,30], which meets our initial expectation. However, it is noted that the lifetime of both the TPBi and Bphen devices is not very long and comparable with some results of fluorescent OLEDs reported by other groups [31,32,33]. Our results suggest that current stressing may induce joule heating, causing accelerated device degradation [19,29,30]. Besides, concentration quenching, often referred to as singlet exciton-charge annihilation, may also exist in our devices with high concentration (20 wt.%) BCzVBi doped in the host [34]. At high concentration, dopant aggregation occurs, facilitating charge injection and transport, while also enhancing exciton interaction and nonradiative decay. What’s more, the stability of host and fluorescent materials used in this study is also an important factor. Further improvement of devices’ lifetime will be conducted in our future research work using other methods such as pulsed current stressing and device structure engineering.

## 4. Conclusions

We have investigated the luminous characteristics of thermal evaporation blue OLEDs with various HTL and ETL materials. Fixing the electron transport material TPBi, four hole transport materials, including NPB, TAPC, CBP, and MoO_3_, were selected to be HTLs. Of these four kinds of devices, the blue OLED with TAPC HTL exhibited a maximum luminance of 2955 cd/m^2^ and current efficiency (CE) of 5.75 cd/A at 50 mA/cm^2^, which are 68% and 62% higher, respectively, than those of the minimum values in the device with the MoO_3_ ETL. This can be explained by the charge balance in the EML. In order to improve the injection and transport of electrons, Bphen was introduced and a maximum luminance of 3640 cd/m^2^ at 50 mA/cm^2^ was obtained, which is 23% higher than that of the TPBi device. Furthermore, the Bphen device showed a half-period of ~3.22 h, which is two times longer than that of the TPBi device stressed under 20 mA/cm^2^. These results indicate that the carrier mobility of transport materials and energy level alignment of different functional layers play important roles in the performance of OLEDs. The findings present a rational strategy for the device engineering of high-efficiency blue OLEDs.
